# Mutation Accumulation in a Selfing Population: Consequences of Different Mutation Rates between Selfers and Outcrossers

**DOI:** 10.1371/journal.pone.0033541

**Published:** 2012-03-20

**Authors:** Shin-Ichiro Nakayama, Shoi Shi, Masaki Tateno, Masakazu Shimada, K. Ryo Takahasi

**Affiliations:** 1 Nikko Botanical Gardens, Graduate School of Science, The University of Tokyo, Tokyo, Japan; 2 Faculty of Science and Technology, Tokyo University of Science, Noda, Chiba, Japan; 3 Department of General Systems Studies, Graduate School of Arts and Science, The University of Tokyo, Tokyo, Japan; 4 Faculty of Life Sciences, Kyoto Sangyo University, Kyoto, Japan; British Columbia Centre for Excellence in HIV/AIDS, Canada

## Abstract

Currently existing theories predict that because deleterious mutations accumulate at a higher rate, selfing populations suffer from more intense genetic degradation relative to outcrossing populations. This prediction may not always be true when we consider a potential difference in deleterious mutation rate between selfers and outcrossers. By analyzing the evolutionary stability of selfing and outcrossing in an infinite population, we found that the genome-wide deleterious mutation rate would be lower in selfing than in outcrossing organisms. When this difference in mutation rate was included in simulations, we found that in a small population, mutations accumulated more slowly under selfing rather than outcrossing. This result suggests that under frequent and intense bottlenecks, a selfing population may have a lower risk of genetic extinction than an outcrossing population.

## Introduction

A large part of spontaneous mutations are deleterious [1], which tend to accumulate continuously in a small population. Such accumulation of deleterious mutations may lead to genetic extinction by lowering the mean fitness of the population [2]. Current theories predict that this accumulation process proceeds much easier in a selfing population [3]. This is because homozygous mutations can never be removed from a selfing lineage, and just like Muller's ratchet in a finite asexual population [4], [5], deleterious mutations accumulate irreversibly. This ratchet process is one of the reasons why a selfing population may have a higher extinction risk than an outcrossing population [2], [6].

So far, the theory has assumed an equal rate of deleterious mutation for both selfers and outcrossers. However, this assumption has not been justified empirically. Analyzing a deterministic model for the evolution of inbreeding in an infinite population, Lande and Schemske [7] found that when the extent of inbreeding depression is lower than 0.5, only complete selfing can be evolutionarily stable, whereas other strategies including outcrossing as well as partial selfing become unstable; conversely, when the extent of inbreeding depression is higher than 0.5, only complete outcrossing can be evolutionarily stable. Furthermore, the mean extent of inbreeding depression was obtained analytically (assuming complete selfing; [8]) and also by simulations [9], which indicated that the deleterious mutation rate, as well as the intensity of selection against deleterious alleles, affects the extent of inbreeding depression. Therefore, extant selfers and outcrossers are expected to have distinct mutational properties: because selfing is evolutionarily stable only when the extent of inbreeding depression is relatively low [7], selfers should retain a deleterious mutation rate that leads to a sufficiently low extent of inbreeding depression; on the contrary, outcrossers should retain a deleterious mutation rate that generates more intense inbreeding depression, because outcrossing is evolutionarily stable only under a higher extent of inbreeding depression [7]. This expected difference has not been reflected in the previous studies that contrasts the rates of mutation accumulation [10] or population extinction [2] under selfing versus outcrossing.

To incorporate the possible difference in deleterious mutation rate between selfers and outcrossers, we analyzed two sets of mathematical models: (i) an analytical model for deriving the rates of deleterious mutation under which selfing and outcrossing, respectively, are evolutionarily stable in an infinite population and (ii) an individual-based model for simulating the process of mutation accumulation in a finite population. We found that when the potential difference in mutation rate (which was predicted from the analytical model) was appropriately accounted for in the simulation model, the speed of mutation accumulation was slower in a small selfing population than an outcrossing population of the same size. In the simulations, we also included the effect of slight outcrossing, which has been shown to slow down the accumulation process in a small selfing population [10], [11]. Assuming 3% of outcrossing in a predominantly selfing population, the range of population size leading to slower mutation accumulation in a selfing population was significantly expanded.

## Analysis

### General model

We largely followed previous formulations [10], [12] and assumed a population of *N* hermaphroditic diploids with no overlapping generations. (Note that *N* refers to the actual size of the population, which may be different from its effective size.) Each individual in the population was assumed to have a fixed number *L* of diallelic loci (or sites), initially carrying no deleterious alleles. Each generation consisted of three stages; mutation, reproduction, then selection. It started from the mutation stage, in which target loci for new deleterious mutations were chosen randomly. The number of target loci per individual followed a Poisson distribution with a mean of 2*U*, where *U* is the deleterious mutation rate per haploid genome per generation. We assumed unidirectional mutation (from wild-type to deleterious alleles) and ignored backward mutation.

In the next reproduction stage, each individual produced a fixed number *m* of seeds (or fertilized eggs in animals) either by random mating (outcrossing) or by selfing. The proportion of selfing was fixed at *S* (0≤*S*≤1, where *S* = 1 indicates complete selfing). When seeds were produced by outcrossing, mating pairs were chosen randomly among distinct individuals within the population.

In the selection stage, surviving seeds were selected with probabilities proportional to their viabilities. The viability of a seed was determined by the numbers of loci in the seed that were heterozygous and homozygous for the deleterious allele, denoted respectively by *i* and *j*. Assuming the multiplicative viability effects of distinct loci, the relative seed viability can be given by (1−*hs*)*^i^*(1−*s*)*^j^*, where *h* and *s* denote the dominance and selection coefficients, respectively. Defined in this way, the relative viability of mutation-free seeds (*i* = *j* = 0) is unity.

### Analytical model

To elucidate the effect of different mutation rates on the level of inbreeding depression, and further to determine the conditions under which selfing and outcrossing become evolutionarily stable, we first investigated the general model analytically by assuming an infinite population (*N* = ∞) and an infinite number of loci (*L* = ∞).

**Figure 1:**
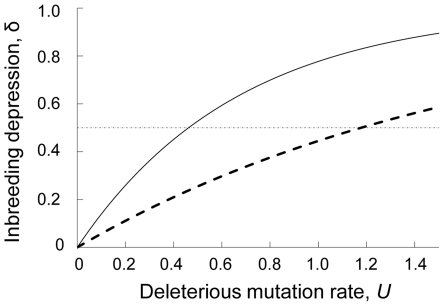
The extent of inbreeding depression as a function of deleterious mutation rate. Mean inbreeding depression under complete outcrossing (*S* = 0; solid line) and complete selfing (*S* = 1; broken line), were obtained analytically for an infinite population (equations 1a, b). The dashed-dotted line shows the threshold level of inbreeding depression ( = 0.5), below which selfing is evolutionarily stable. Selection parameters are set to be *h* = 0.2 and *s* = 0.1, based on available data for *Drosophila melanogaster* [19], [20].

We analyzed two distinct cases separately, assuming either complete outcrossing (*S* = 0) or complete selfing (*S* = 1). Based on recursion relations for the mean numbers of loci in an individual that are either heterozygous or homozygous for a deleterious allele (see Text S1 and Table S1), we analytically obtained the equilibrium level of inbreeding depression (*δ*; see equation A3 in Text S1 for definition) as
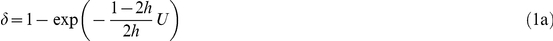
(1a)for a completely outcrossing population, and
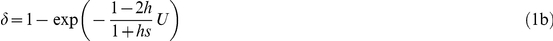
(1b)for a completely selfing population. The dependence of *δ* on the genomic rate of deleterious mutation (*U*) under *s* = 0.01 and *h* = 0.2 is depicted in Figure 1, which clearly shows that for a given value of *U*, an outcrossing population typically retains a higher extent of inbreeding depression [as long as *h*<1/(2−*s*); see equations 1].

We then derived conditions under which selfing and outcrossing could be evolutionarily stable. Lande and Schemske [7] suggested that selfing is evolutionarily stable (and outcrossing is evolutionarily unstable) when *δ*<0.5 (although they did not consider the variance among individuals in the number of mutations). We see from equation (1a) that when
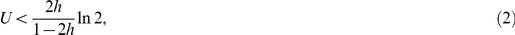
(2)inbreeding depression falls below the suggested threshold of 0.5, even in an outcrossing population where a higher extent of inbreeding depression is expected (see also Figure 1). This implies that assuming *h* = 0.2 as in Figure 1, the sufficient condition for the evolution of selfing is *U*<0.46. Equations (1) also suggest that when
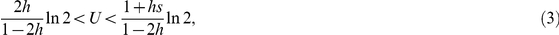
(3)selfing and outcrossing are bistable, meaning that populations monomorphic for either selfing or outcrossing can both be evolutionarily stable. Lastly when
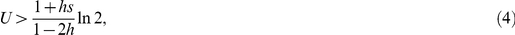
(4)only outcrossing is stable.

Based on these results, we predict that extant selfers maintain lower deleterious mutation rates [satisfying conditions (2) or (3)], whereas in outcrossers, the rates are higher [characterized by conditions (3) or (4)].

### Simulation model

To investigate the speeds of mutation accumulation in finite populations with different rates of selfing, we conducted a series of individual-based stochastic simulations, assuming *N*<∞, *L* = 20,000, *m* = 20, and *h* = 0.2. Again, mutation was assumed unidirectional. Because the number of loci was assumed finite, apparently no new mutations were introduced at a randomly chosen locus if it was already homozygous for the deleterious allele. On the other hand, if the locus was homozygous for the wild-type allele, it then became a heterozygote. If the chosen locus harbored a single copy of the deleterious allele as a heterozygote, then with a probability of 0.5, it became homozygous for the deleterious allele; otherwise, it remained heterozygous. Thus, as deleterious mutations accumulated, the effective rate of overall mutation became smaller. However, because the proportion of mutated loci remained small in the present analysis, this decrease in the effective mutation rate was only of minor importance. Hence, the deleterious mutation rate was always kept close to the original level of 2*U* per diploid genome.

**Figure 2:**
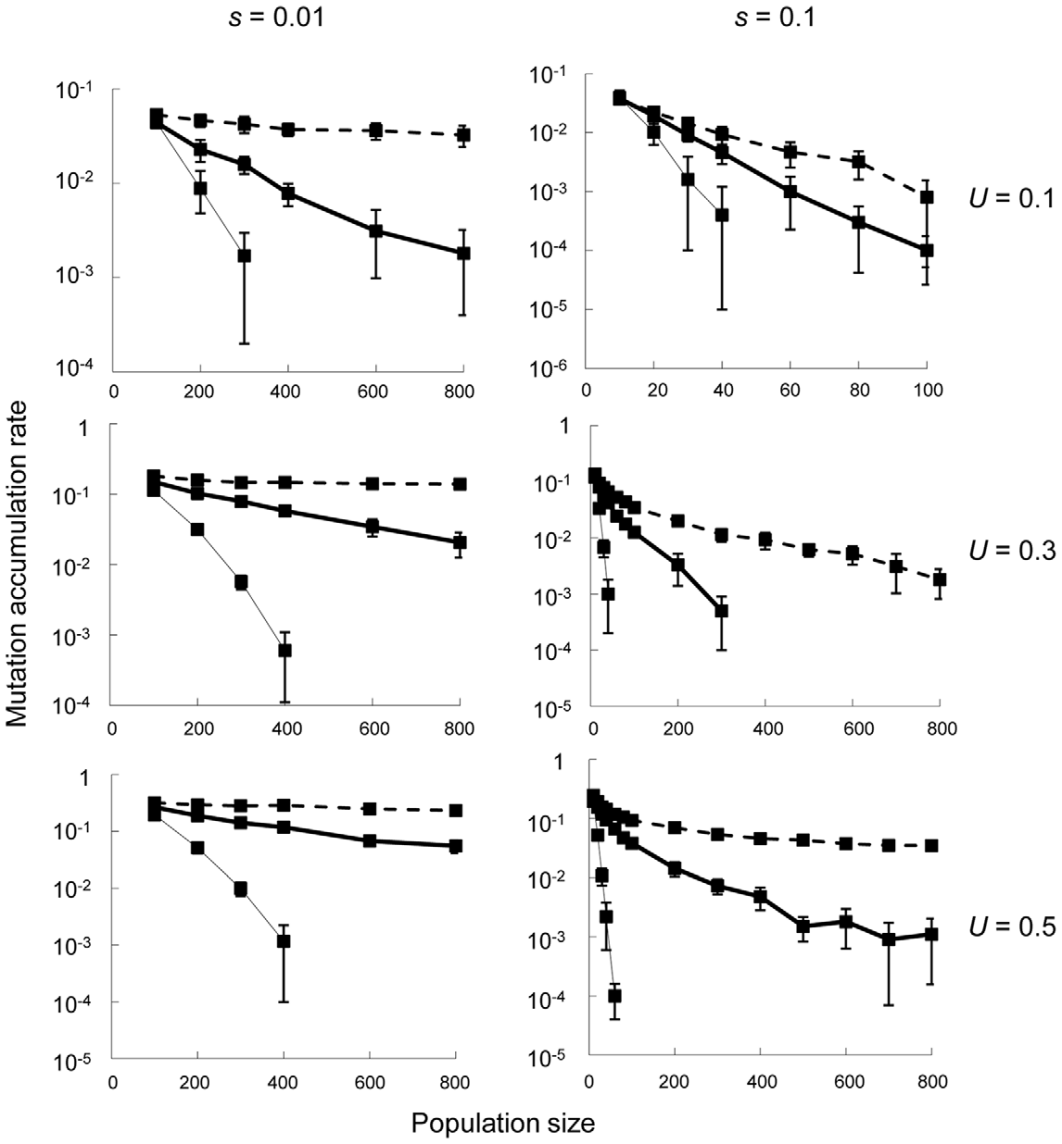
The rate of mutation accumulation as a function of population size. For each combination of *U* ( = 0.1, 0.3, and 0.5) and *s* ( = 0.01 and 0.1), simulation results are illustrated for populations under complete selfing (*S* = 1; broken lines), predominant selfing with slight outcrossing (*S* = 0.97; heavy solid lines), or complete outcrossing (*S* = 0; thin solid lines). The average rate of mutation accumulation was obtained by taking the arithmetic mean over 1000 generations after the systems reached steady states. Error bars indicate the standard deviations. Throughout, the degree of dominance was *h* = 0.2. The data points for situations with no fixation (i.e. when the mutation accumulation rate was zero) were omitted from the figure. Note that ordinates are given in (common) logarithmic scale. Note also that the ranges of parameters shown in the two axes vary among panels.

Every generation the mean numbers of loci in an individual that were heterozygous and homozygous for the deleterious allele were recorded, along with the number of deleterious alleles fixed in the population. In each run of simulations, we observed that the number of heterozygous loci always reached a certain equilibrium, whereas the number of loci with homozygous mutations kept gradually increasing (Figure S1). This increase in the number of homozygous loci always proceeded at an approximately constant rate, which was roughly equivalent to the average rate of fixation, defined here as the average increase per generation in the number of deleterious alleles being fixed in the population. This observation clearly indicates that in our simulations, mutation accumulation was caused by recurrent fixation of deleterious alleles, not by an increase in the number of deleterious alleles that were still segregating in the population. In the following, we adopted the average fixation rate as the measure to quantify the speed of mutation accumulation. All simulations were iterated 10 times for each set of *U* ( = 0.1, 0.3, or 0.5), *s* ( = 0.1 or 0.01), and *S* ( = 0, 0.97, or 1) (Figure 2). We were particularly interested in comparing the speeds of mutation accumulation in selfing and outcrossing populations, assuming distinct rates of deleterious mutation as predicted from the analytical model; say, *U* = 0.1 or 0.3 for selfers (*S* = 0.97 or 1), and *U* = 0.5 for outcrossers (*S* = 0). According to our analytical results for an infinite population [see conditions (2)–(4) above], *U* = 0.5 corresponds approximately to the lowest value appropriate for outcrossers, just above the threshold rate of *U* = 0.46 for the evolutionary instability of outcrossing (assuming *h* = 0.2). In contrast, *U* = 0.1 and 0.3 meet the criteria for the evolution of selfing. Although selfing can be evolutionarily stable also when *U*>0.46 [as long as condition (3) is met], we here restricted our attention to smaller values of *U* (0.1 and 0.3) for selfers, which satisfy the more stringent condition (2). Given that outcrossing is ancestral to selfing in most cases [13], [14], we expect that most populations remain outcrossing when *U*>0.46, because under this condition, outcrossing can also be evolutionarily stable and the transition from outcrossing to selfing is unlikely.

**Figure 3:**
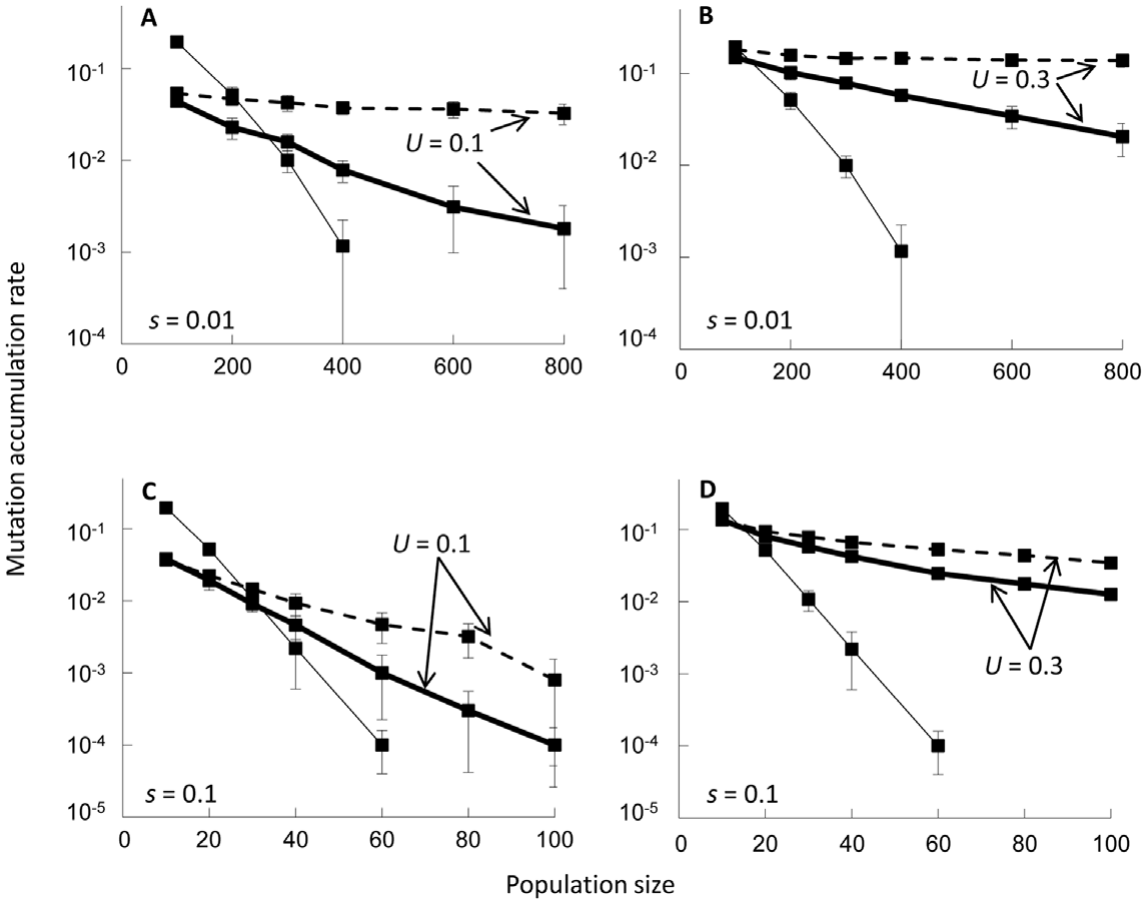
Comparison of the mutation accumulation rates when deleterious mutation rates vary between mating systems. Simulation results are illustrated for populations under complete selfing (*S* = 1; broken lines), predominant selfing with slight outcrossing (*S* = 0.97; heavy solid lines), or complete outcrossing (*S* = 0; thin solid lines). The selection coefficient was either *s* = 0.01 (A, B) or 0.1 (C, D), with *h* = 0.2 in all cases. Distinct rates of deleterious mutation were assigned to selfing and outcrossing populations, for which appropriate mutation rates were predicted from the analytical model as developed in Text S1. The mutation rate was kept at *U* = 0.1 (A, C) or 0.3 (B, D) for populations with complete selfing or slight outcrossing, whereas *U* = 0.5 for completely outcrossing populations. Error bars indicate the standard deviations. Note that ordinates are given in (common) logarithmic scale. The data points for situations with no fixation (i.e. when the mutation accumulation rate was zero) were omitted from the figure. Note also that the range of population size shown in the horizontal axis varies among panels.

When distinct rates of deleterious mutation were appropriately assigned to selfing and outcrossing populations, mutation accumulation proceeded more slowly in a selfing rather than an outcrossing population if the population size was very small (Figure 3). For example, under weak selection (*s* = 0.01), we found that roughly when *N*<200, mutations accumulated more slowly in a completely selfing population (with *U* = 0.1) than a completely outcrossing population (with *U* = 0.5) (Figure 3A). Only 3% of outcrossing considerably retarded the rate of mutation accumulation regardless of the strength of selection (Figures 2 and 3). We further found that the range of population size leading to slower mutation accumulation in a selfing population was wider when slight outcrossing was performed, and also when the strength of selection was reduced (Figure 3).

## Discussion

### Extinction risk of a selfing population

In this study, we theoretically examined the effect of the predicted difference in deleterious mutation rate between selfers and outcrossers on the speeds of mutation accumulation under the two mating systems. We found that when the population size was sufficiently small, the difference in mutation rate generated specific situations under which mutation accumulation proceeded more slowly in a selfing than an outcrossing population (Figure 3). These situations arise because in a small population, consanguineous matings occur even under random mating, and the difference among mating systems in ability to purge deleterious mutations would be minimal (Figure 2). The advantage of selfers having low deleterious mutation rates would then be manifested (Figure 3), eventually leading to slower mutation accumulation in a selfing population.

Furthermore, a reduction in selection intensity expanded the range of population size favorable for selfing (Figure 3). Unfortunately, not much is known empirically about the distribution of fitness effects among new mutations [1], whether the species are selfing or outcrossing. However, Davies *et al.* [15] found that in *Caenorhabditis elegans*, more than 96% of mutations have extremely small fitness effects (*s*<0.0007). If such small values of *s* are the rule rather than exceptions, the range of population size favorable for selfing might be wider than previously thought.

We also found that only 3% of outcrossing was sufficient to considerably retard the rate of mutation accumulation, thereby expanding the range of population size favorable for selfing (Figure 3). Although many species are known to retain extremely high selfing rates, it has also been found that many of them are still capable of low rates of outcrossing. For example, among 55 plant species studied by Schemske and Lande [16], 23 were categorized as predominantly selfing (those with selfing rates over 0.8), but only a single lineage was found to be a complete selfer (with the selfing rate of 1). Others were performing low but non-negligible levels of outcrossing (∼3%, estimated as an approximate average for species with selfing rates over 0.9; [16]). More recent estimates also follow a similar tendency [17]. It should be noted that the retardation effect of slight outcrossing has previously been observed in simulations of relatively small populations (100 individuals or less; [10], [11]), but in our analysis, this effect was evident in rather large populations (Figures 2 and 3).

These findings lead us to speculate that if a population is forced to go through frequent bottlenecks, the risk of extinction by genetic degradation might be lower under selfing than outcrossing. As shown by François *et al.* [18], a predominantly selfing weed *Arabidopsis thaliana* has been successful at colonizing unoccupied lands that have been disrupted by human activities. It is expected that during such colonization events, both selfers and outcrosses experience a similar amount of reduction in population size, eventually giving rise to a situation favorable for selfing (see above). Thus, in highly disturbed habitats, the establishment of selfing populations would be much facilitated, although it is influenced also by many other ecological factors, such as the relative advantage of selfing in acquiring mates under extremely low densities.

### Do selfers have lower deleterious mutation rates?

We have seen that values of *U* and *h* have profound effects on the extent of inbreeding depression (equations 1, Figure 1), which may in turn affect the direction of mating system evolution. Based on a classical estimate for *Drosophila melanogaster* [19], [20], we assumed a fixed value of *h* ( = 0.2) throughout. In plants, there are a few estimates of *h* for nonlethal detrimental mutations: *h* = 0.15 in *Mimulus guttatus* [21], *h* = 0.28 and 0.35 in *Amsinckia gloriosa* [22], and *h* = 0.14 and 0.069 in *A. spectabilis* [22]. Despite such variation in *h*, there should still be a certain threshold of *U* in each species, below which the evolution of selfing would be promoted (as exemplified for *h* = 0.2 in Figure 1). This is because the extent of inbreeding depression monotonically increases with an increase in *U* as long as deleterious mutations are partially recessive (0<*h*<0.5 in equations 1; see also Figure 1).

It has been shown that outcrossing was selected in mutagen-treated populations of *C. elegans* [23], suggesting that outcrossing is selectively favored under high mutation pressure. This result seems to support, albeit indirectly, the plausibility of our expectation that selfers should have lower deleterious mutation rates. It was also suggested recently that selfing might directly reduce the rate of mutation [24]. Although this possibility was not considered in the present analysis, it can further exaggerate the putative difference in deleterious mutation rate between selfers and outcrossers. Then, after all, do selfers retain lower deleterious mutation rates than outcrossers?

Although estimates of *U* are available for only a handful of species, limited data across diverse taxa suggest that selfers indeed have lower rates of deleterious mutation: e.g. *U* = 0.07–0.1 (*A. thaliana*; [25]) and *U* = 0.48 (*C. elegans*; [26]) for predominantly selfing species; *U* = 0.6 (*D. melanogaster*; [27]) and *U*>1.5 (humans; [28]) for outcrossing species. In contrast, *U* did not differ significantly between closely related species of nematodes with different mating systems [29]. Although this result does defy our expectation that selfers should have lower deleterious mutation rates, our analytical considerations suggest several possibilities: (i) the degree of dominance (*h*) is higher in selfers, so that the inbreeding depression falls below 0.5 only in selfers; or (ii) selfers and outcrossers share the same mutational properties, under which the necessary condition (3) for the evolution of selfing can only be fulfilled. In case (ii), because both outcrossing and selfing are evolutionarily stable, a stochastic change in the selfing rate may eventually lead to an evolutionary shift in the mating system. To confirm these predictions, it is essential to obtain a precise estimate for the (average) degree of dominance in each species. More importantly, there are still too few data available for directly testing the assumed difference in deleterious mutation rate between selfing and outcrossing species. Such verification would be crucial for properly understanding how and when selfing promotes the extinction of natural populations.

## Supporting Information

Text S1 **Supplementary analysis.**
(DOC)

Figure S1 **Dynamics of mutation accumulation.** The temporal changes in the number of loci that were heterozygous (thin solid lines), homozygous (but not fixed, heavy solid lines), or fixed (broken lines) for deleterious alleles are illustrated. The averages obtained from 10 simulation runs are shown for a completely outcrossing population (top) and a completely selfing population (bottom). Other parameters are *U* = 0.3, *s* = 0.01, and *N* = 200. Note that the average number of heterozygous loci in the selfing population was so small (<1) that it is essentially superimposed onto the horizontal axis.
(TIF)

Table S1 **Mean number of mutated loci per individual at each stage.**
(PDF)
