# Supplementary material for: Mutation Accumulation in a Selfing Population: Consequences of Different Mutation Rates between Selfers and Outcrossers
Source: PLoS One. 2012 Mar 20;7(3):e33541. doi: 10.1371/journal.pone.0033541 (PMC3308984; doi:10.1371/journal.pone.0033541)
Supplement: Text S1 — Supplementary analysis. (DOC) [file pone.0033541.s001.doc]

**Supporting Information**

**Text S1**

**Basic recursions**

We studied the changes over time in the frequency of individuals carrying different numbers of mutations. The frequency at the beginning of the *n*-th generation is designated by *P_n_*(*x*, *y*), where *x* and *y* denote the numbers of loci in an individual that are heterozygous and homozygous for the deleterious allele, respectively. The frequency immediately after the mutation stage is given by

$P'_{n}(x,y)=\sum_{i=0}^{x} P_{n}(i,y)Q_{2U}(x-i)$,

where *Q*_2_*_U_*(.) denotes the Poisson distribution with mean 2*U*;

$Q_{2U}(x)=\frac{e^{-2U}(2U)^{x}}{x!}$.

Because we assume infinitely many loci, new mutations are always introduced as heterozygotes, and the number *y* of homozygous loci is not altered during the mutation stage. Therefore, *P_n_*'(*x*, *y*) is obtained by taking the sum of probabilities that *x – i* mutations are newly introduced to individuals with *i* heterozygous and *y* homozygous loci. After meiosis, the frequency of haploid gametes carrying *x* deleterious mutations becomes

.

Because *j* mutations are automatically inherited from the *j* homozygous loci, *p_n_*(*x*) can be obtained as the sum of probabilities that *x* – *j* mutations are inherited from the *i* heterozygous loci. Using *p_n_*(*x*) and assuming random union of gametes, the frequency of diploid individuals after outcrossing in the reproduction stage is described as

$P_{n}^{(\text{O})}(x,y)=\sum_{i=0}^{x} p_{n}(i)p_{n}(x-i)Q_{0}(y)$,

which is the sum of probabilities that a gamete carrying *i* mutations fertilizes a gamete carrying *x – i* mutations. Again, because we assume an infinite number of loci, the possibility that mutations become homozygotes can be ignored; hence the Poisson distribution with mean 0 (*Q*_0_ ) appears in the above equation. Consequently, after outcrossing, the number of homozygous mutations is zero;

$P_{n}^{(\text{O})}(x,0)=\sum_{i=0}^{x} p_{n}(i)p_{n}(x-i)$ and

$P_{n}^{(\text{O})}(x,y)=0$ for *y* ≠ 0.

Likewise, the frequency of diploid individuals after selfing in the reproductive stage is described as

(see also [1], p. 640).

Using the above annotations, the frequency of diploid individuals after the reproduction stage is described as

$P''_{n}(x,y)=(1-S)P_{n}^{\text{(O)}}(x,y)+SP_{n}^{\text{(S)}}(x,y)$,

where *S* denotes the selfing rate. Finally, the frequency after selection in the next generation is described as

$P_{n+1}(x,y)=\frac{1}{K_{n}}(1-hs)^{x}(1-s)^{y}P''_{n}(x,y)$,

where *K_n_* is the population mean viability.

Based on this recursion relation, we analytically investigated the two extreme cases, with *S* = 0 (complete outcrossing) or *S* = 1 (complete selfing). Initially, the frequencies of diploid individuals were determined by assuming that the number of heterozygous loci follows a Poisson distribution with mean *v*_0_, whereas for homozygous loci, it is given by a Poisson distribution with mean *w*_0_. Hence, we have

$P_{0}(x,y)=Q_{v_{0}}(x)Q_{w_{0}}(y)$.

**Mean numbers of loci with heterozygous and homozygous mutations**

It is easily shown that the number of heterozygous loci per individual follows a Poisson distribution at every stage in every generation, once it is Poisson-distributed. This also holds for the number of loci that are homozygous for the deleterious allele. Since a Poisson distribution has only a single parameter (i.e. its mean), the entire system can be determined solely by the mean values (here denoted *v_n_* and *w_n_*, respectively) of the two Poisson-distributed variables. Table S1 lists the mean values at each stage in a life cycle. Hence, their changes through generations are described by

$$v_{n+1}=(1-hs)(v_{n}+2U)$$

with $w_{n}=0$ for outcrossing, and

$v_{n+1}=(1-hs)(\frac{v_{n}}{2}+U)$ and

$$w_{n+1}=(1-s)(w_{n}+\frac{v_{n}}{4}+\frac{U}{2})$$

for selfing.

Based on these recursion relations, the mean number of heterozygous loci under complete outcrossing (*S* = 0) is obtained as

$v_{n}=(1-hs)^{n}\left\{v_{0}-\frac{2(1-hs)}{hs}U\right\}+\frac{2(1-hs)}{hs}U$;

at equilibrium (*n* = ∞), this becomes

$v_{\infty}=\frac{2(1-hs)}{hs}U$. (A1)

Note that $w_{n}=w_{\infty}=0$ always holds for the homozygous loci.

Under complete selfing (*S* = 1), the mean numbers of loci with heterozygous and homozygous mutations become

$v_{n}=\left(\frac{1-hs}{2}\right)^{n}\left\{v_{0}-\frac{2(1-hs)}{1+hs}U\right\}+\frac{2(1-hs)}{1+hs}U$ and

$$\begin{matrix} w_{n}=\left\{\left(\frac{1-hs}{2}\right)^{n}-(1-s)^{n}\right\}\frac{(1-s)v_{0}}{2(2s-hs-1)}+(1-s)^{n}w_{0} \\ -\frac{1-s}{2s-hs-1}U\left\{\frac{1-hs}{1+hs}\left(\frac{1-hs}{2}\right)^{n}-\frac{(1-s)^{n+1}}{s}\right\}+\frac{1-s}{s(1+hs)}U, \end{matrix}$$

which respectively become

$v_{\infty}=\frac{2(1-hs)}{1+hs}U$ and (A2a)

$w_{\infty}=\frac{1-s}{s(1+hs)}U$ (A2b)

as *n* → ∞. Because these equilibria are globally stable, the system eventually reaches either equilibrium (depending on whether *S* = 0 or 1) regardless of the initial state.

**Mean viability and inbreeding depression**

Using the equilibrium numbers (A1) or (A2), any quantity at equilibrium can be obtained. The equilibrium mean viability, which is defined as

$K_{\infty}=\sum_{i=0}^{\infty} \sum_{j=0}^{\infty} (1-hs)^{i}(1-s)^{j}P_{\infty}(i,j)$,

becomes

$$K_{\infty}=\exp\left(-2U\right)$$

under complete outcrossing, and

$$K_{\infty}=\exp\left(-\frac{1+2hs}{1+hs}U\right)$$

under complete selfing. The equilibrium mean viability under complete outcrossing, which does not depend on the selection parameters *h* and *s*, recovers the Haldane-Muller principle [2]. In a similar manner, inbreeding depression, defined as

$\delta=1-\frac{\text{mean viability of selfed seeds}}{\text{mean viability of outcrossed seeds}}$, (A3)

can also be obtained as equations (1a) and (1b) in the text. These analytical findings are consistent with the results obtained previously [3, 4].

**References**

1. Kondrashov AS (1985) Deleterious mutation as an evolutionary factor. II. Facultative apomixes and selfing. *Genetics*, 111: 635-653.
2. Haldane JBS (1957) The cost of natural selection. *J. Genet.*, 55: 511-524
3. Charlesworth B, Charlesworth D, and Morgan MT (1990) Genetic loads and estimates of mutation rates in highly inbred plant populations. *Nature*, 347: 380-382.
4. Charlesworth D, Morgan MT, and Charlesworth B (1990) Inbreeding depression, genetic load, and the evolution of outcrossing rates in a multilocus system with no linkage. *Evolution*, 44: 1469-1489.
