# Supplementary figures and images for: Mutation Accumulation in a Selfing Population: Consequences of Different Mutation Rates between Selfers and Outcrossers
Source: PLoS One. 2012 Mar 20;7(3):e33541. doi: 10.1371/journal.pone.0033541 (PMC3308984; doi:10.1371/journal.pone.0033541)

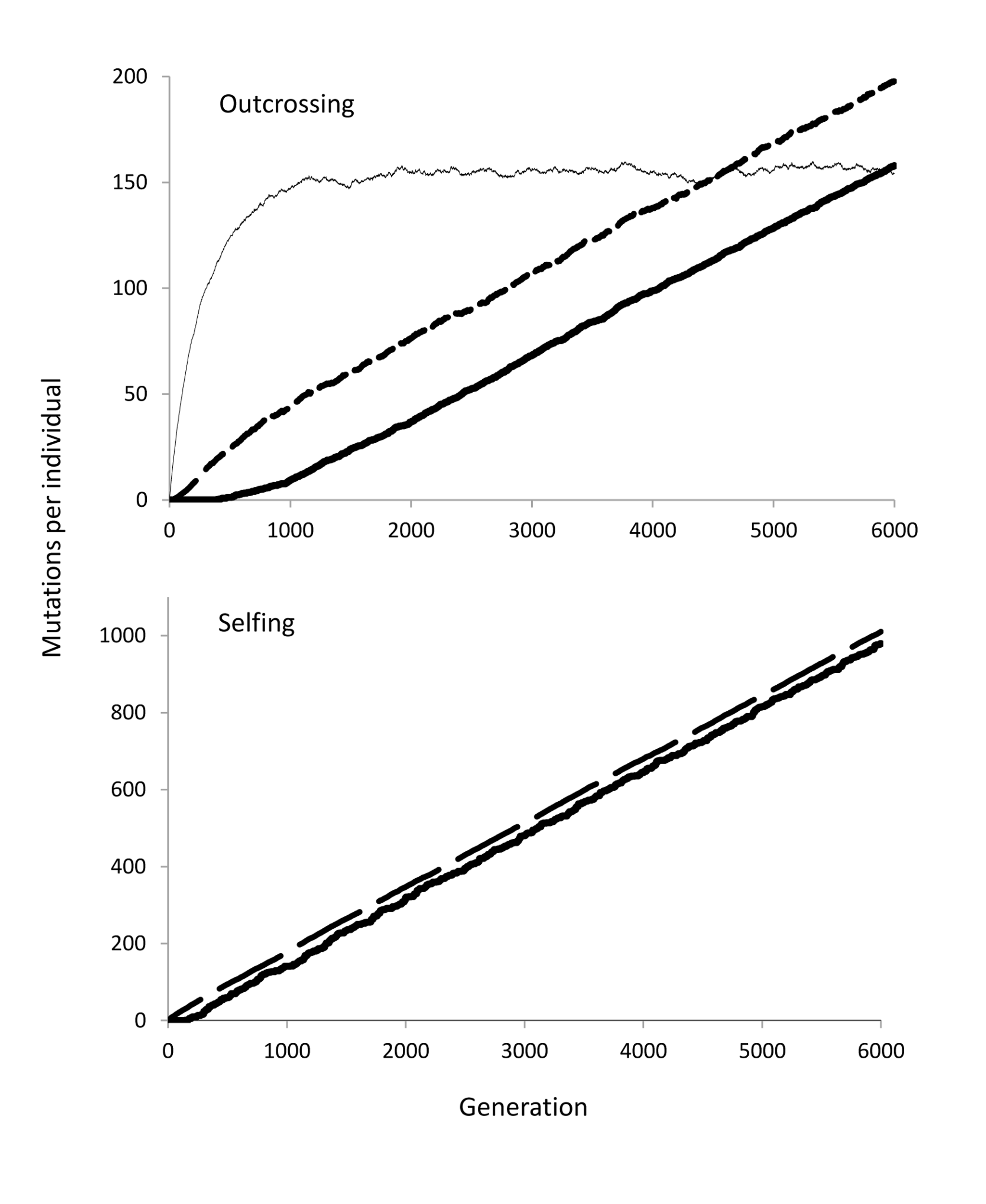

Supplement: Figure S1 — Dynamics of mutation accumulation. The temporal changes in the number of loci that were heterozygous (thin solid lines), homozygous (but not fixed, heavy solid lines), or fixed (broken lines) for deleterious alleles are illustrated. The averages obtained from 10 simulation runs are shown for a completely outcrossing population (top) and a completely selfing population (bottom). Other parameters are U = 0.3, s = 0.01, and N = 200. Note that the average number of heterozygous loci in the selfing population was so small (<1) that it is essentially superimposed onto the horizontal axis. (TIF) [file pone.0033541.s002.tif]
