# Supplementary material for: Mutation Accumulation in a Selfing Population: Consequences of Different Mutation Rates between Selfers and Outcrossers
Source: PLoS One. 2012 Mar 20;7(3):e33541. doi: 10.1371/journal.pone.0033541 (PMC3308984; doi:10.1371/journal.pone.0033541)
Supplement: Table S1 — Mean number of mutated loci per individual at each stage. (PDF) [file pone.0033541.s003.pdf]

Table S1. Mean number of mutated loci per individual at each stage in the n-th generation.

	Outcrossing		Selfing	
Stage	Heterozygous loci	Homozygous loci	Heterozygous loci	Homozygous loci
Mutation	$v_n + 2U$	0	$v_n + 2U$	w_n
Reproduction	$v_n + 2U$	0	$v_n/2 + U$	$w_n + v_n/4 + U/2$
Selection	$(1 - hs)(v_n + 2U) = v_{n+1}$	0	$(1 - hs)(v_n/2 + U) = v_{n+1}$	$(1 - s)(w_n + v_n/4 + U/2) = w_{n+1}$

Initially before mutation, the mean numbers of loci that are heterozygous and homozygous for the deleterious allele are given by v_n and w_n, respectively.
